# Prediction of essential proteins based on subcellular localization and gene expression correlation

**DOI:** 10.1186/s12859-017-1876-5

**Published:** 2017-12-01

**Authors:** Yetian Fan, Xiwei Tang, Xiaohua Hu, Wei Wu, Qing Ping

**Affiliations:** 1 School of Mathematics, Liaoning University, Shenyang, 110036 China; 2 Department of Information Science and Engineering, Hunan First Normal University, Changsha, 410205 China; 3 College of Computer, National University of Defense Technology, Changsha, 410073 China; 4 College of Computing and Informatics, Drexel University, Philadelphia, 19104 USA; 5 School of Mathematical Sciences, Dalian University of Technology, Dalian, 116023 China

**Keywords:** Essential proteins, Subcellular localization information, Modified PageRank algorithm, Protein-protein interaction networks

## Abstract

**Background:**

Essential proteins are indispensable to the survival and development process of living organisms. To understand the functional mechanisms of essential proteins, which can be applied to the analysis of disease and design of drugs, it is important to identify essential proteins from a set of proteins first. As traditional experimental methods designed to test out essential proteins are usually expensive and laborious, computational methods, which utilize biological and topological features of proteins, have attracted more attention in recent years. Protein-protein interaction networks, together with other biological data, have been explored to improve the performance of essential protein prediction.

**Results:**

The proposed method SCP is evaluated on Saccharomyces cerevisiae datasets and compared with five other methods. The results show that our method SCP outperforms the other five methods in terms of accuracy of essential protein prediction.

**Conclusions:**

In this paper, we propose a novel algorithm named SCP, which combines the ranking by a modified PageRank algorithm based on subcellular compartments information, with the ranking by Pearson correlation coefficient (PCC) calculated from gene expression data. Experiments show that subcellular localization information is promising in boosting essential protein prediction.

## Background

Although essential proteins are only a small fraction of all proteins, they are indispensable to maintain life for an organism [1, 2]. Without these essential proteins providing all available nutrients [3], it will lead to lethality of life. Therefore, reliable identification of essential proteins is significant for biologists, for that it not only contributes to understanding the basic requirements for subcellular survival, but also plays a key role in practical implications, such as diseases analysis [4, 5], drug design [6, 7] and medical treatments [4]. This problem has attracted enormous amount of researchers, and many experimental methods have been proposed to predict and discover essential proteins through gene knock-out [8, 9], gene knockdown [10–12] and RNA interference [13]. These methods can provide an accurate prediction of essential proteins. However, the poor efficiency and high cost of experimental methods remains a significant challenge. In addition, for identification of essential proteins in some complex organisms, especially ones from humans, these experimental methods are not suitable.

To break through these experimental constraints, some researchers proposed computational methods to predict essential proteins based on features developed in experimental studies. Especially, due to the high-throughput techniques, abundant data of essential proteins has been collected, which served as the basis for several studies that investigate the relationship between characteristics of experimentally identified essential proteins and their topological properties in protein-protein interaction networks (PPI). With the help of computational methods, the burden to test all proteins in experiments can be greatly relieved, so that only tests of top-ranked proteins based on their score of essentiality are prioritized. Jeong et al. used centrality-lethality rule to identify essential proteins in protein-protein interaction networks, which means that proteins most highly connected in the networks tend to be essential proteins [14]. Pereira-Leal et al. reported that there is higher-level correlation among essential proteins compared to that among nonessential proteins [15]. To explain this phenomenon, He and Zhang proposed the concept of essential protein-protein interactions [16]. These studies support the view that evolution of essential PPI networks are more conservative than nonessential PPI networks. Inspired by these studies that explored topological features of PPI networks, some researchers proposed computational methods to identify essential proteins, based on metrics such as betweenness centrality (BC) [17, 18], degree centrality (DC) [19], edge clustering coefficient centrally (NC) [20] and so on. However, all these methods relying on centrality metrics share some limitations. First, PPI networks generated by high-throughput technologies are often incomplete and contain false positive interactions [21]. Second, many of these methods neglect other intrinsic properties of essential proteins. To overcome these limitations, several methods are proposed to incorporate these PPI networks with other biological data. Based on the weighted PPI networks generated by gene expression profiles, Li et al. proposed an edge-aided approach named PeC to predict essential proteins [22]. Then Tang et al. proposed a modified approach named as WDC to improve the prediction performance [23].

Moreover, recently many studies found that the subcellular localization of proteins may play an important role in identifying essential proteins. Acencio and Lemke discover that integration of information from multiple sources including subcellular localization of proteins can improve the accuracy of essential proteins prediction [24]. Peng et al. proposed a Compartment Importance Centrality (CIC) method [25] that incorporate the subcellular localization information in PPI networks. One limitation of CIC method is that it may not differentiate varieties of the interactions among proteins of a large community. To overcome this limitation, in this paper, we propose a novel method that combines information of subcellular compartments with that of Pearson Correlation coefficient (SCP), based on weighted PPI networks to predict essential proteins. Additionally, a modified PageRank method is proposed to assign weights in the PPI networks more accurately.

This paper is organized into four sections. Our algorithm is presented in “Methods” section. Numerical experiments and results analysis are described in “Results and discussion” section. Several conclusions are drawn in “Conclusion” section.

## Methods

In this section, we will present our method SCP, that can rank the importance of proteins with computed scores. The final importance scores of our SCP method is determined by two components: the results ranked by our modified PageRank algorithm (MPR) from subcellular localization information, and the results ranked by Pearson correlation coefficient (IPCC) from gene expression data:

1 $$ \text{SCP} = \lambda \cdot \text{NIS}(\text{MPR}) + (1 - \lambda) \cdot \text{NIS}(\text{IPCC}), \quad \lambda \in [0,1]  $$

where *λ* is an adjusting parameter for weighting the two components. In this paper the parameter *λ* is set as 0.5. The MPR is the importance scores computed from modified PageRank algorithm. The IPCC is the importance scores predicted by Pearson Correlation coefficient. In order to predict essential proteins, we propose a novel algorithm combining MPR with IPCC. We expect that protein with a higher SCP score would be more likely to be an essential protein. As the scores of MPR and IPCC may have different range, they should be scaled into [0,1] first. We normalize the two importance scores as follows:

2 $$ \begin{aligned} \text{NIS} (Score_{i}) &= \frac{Score_{i} - \text{min}(Score) }{ \text{max}(Score) - \text{min}(Score) }, \\ i &= 1,2,\cdots,N \end{aligned}  $$

### MPR importance score of proteins

We first create a weighted PPI networks derived from subcellular compartments information, and then perform a modified PageRank algorithm on the network to compute importance score of proteins. For most eukaryotes, the subcellular compartments generate a specific environment that regulates the biological processes of proteins within cells. Therefore, knowing the subcellular localization of proteins may shed light on understanding the functions of these proteins. Many studies found that proteins interactions in vivo tend to co-locate in the same cellular compartment or adjacent compartments [26]. For example, 76 percent of protein-protein interactions in yeast cells are carried out in the same subcellular compartments [27]. Therefore it may be beneficial to weigh the protein-protein interactions by subcellular localization, and then predict the importance of proteins based on the weighted protein-protein interactions.

Based on this intuition, we develop a metric to weigh the protein-protein interactions based on the information of subcellular localization. We assume that protein-protein interactions co-located in a small subcellular compartment can be more reliable in predicting essential proteins than those within a large subcellular compartment.

#### The importance of subcellular compartments

We model the importance of subcellular compartments based on their scales. Suppose there are K subcellular compartments *C*_1_,*C*_2_,⋯,*C*_*K*_, and the numbers of them are $N_{C_{1}}, N_{C_{2}}, \cdots, N_{C_{K}}$ respectively. Then the importance of subcellular compartment *C*_*i*_, denoted by ISC, is defined as:

3 $$ \text{ISC} (C_{i}) = \frac{1}{N_{C_{i}}}, i = 1,2, \dots, K  $$

#### The weight of protein-protein interactions based on subcellular compartments

The importance of protein-protein interactions can be impacted by different subcellular compartments they share. For a given protein *P*_*i*_, let SCL(*P*_*i*_) be the subcellular compartments where protein *P*_*i*_ located. The weight of *P*_*i*_ and *P*_*j*_ interaction is denoted by W_PPI_(*P*_*i*_,*P*_*j*_), which is defined as:

4 $$ \begin{aligned} &\mathrm{W}_{\text{PPI}}\left(P_{i},P_{j}\right) \\ &=\left\{ \begin{array}{cc} \underset{C_{i} \in \text{SC}(P_{i},P_{j})}{\text{max}}\{\text{ISC}(C_{i})\},& \text{SCL}(P_{i}) \bigcap \text{SCL}(P_{j}) \neq \emptyset,\\ \underset{C_{i} \in \text{SC}(P_{i},P_{j})}{\text{min}}\{\text{ISC}(C_{i})\}, & \text{otherwise} \end{array} \right. \end{aligned}  $$

where

5 $$ \begin{aligned} &\text{SC}\left(P_{i},P_{j}\right) \\ &= \left\{ \begin{array}{cc} \text{SCL}(P_{i}) \bigcap \text{SCL}(P_{j}),& \text{SCL}(P_{i}) \bigcap \text{SCL}(P_{j}) \neq \emptyset,\\ \text{SCL}(P_{i}) \bigcup \text{SCL}(P_{j}), & \text{otherwise} \end{array} \right. \end{aligned}  $$

A pair of proteins may be co-located in several subcellular compartments because many proteins are annotated by multiple subcellular compartments. Here $\text {SCL}(P_{i}) \bigcap \text {SCL}(P_{j})$ means the common subcellular compartments that protein *P*_*i*_ and *P*_*j*_ are co-located in. We assume that a pair of proteins in the smaller subcellular compartments is most likely to interact with each other than them in the bigger compartments. Therefore, if a pair of proteins are co-located in at least one subcellular compartment, that is $\text {SCL}(P_{i}) \bigcap \text {SCL}(P_{j}) \neq \emptyset $, we choose the maximum of the importance of their common subcellular compartments as the importance of the protein-protein interaction between the two proteins. Otherwise, the importance between a pair of proteins which do not share any subcellular compartments will be the minimum of all their subcellular compartments, defined as $\text {SCL}(P_{i}) \bigcup \text {SCL}(P_{j})$.

#### The importance of proteins

By analyzing the weighted protein-protein interaction network, we can achieve prior estimate on the importance of each protein. The proteins which have stronger interactions with others to be more important proteins (essential proteins). Guided by this idea, we sum up all the weights of protein-protein interactions related to a protein *P*_*i*_ as its prior importance (denoted by IPSC(*P*_*i*_)):

6 $$ \text{IPSC} (P_{i}) = \sum_{P_{j} \in \text{SCL}(P_{i})} W_{\text{PPI}}\left(P_{i},P_{j}\right)  $$

### Modified PageRank algorithm

PageRank is one of the most famous methods that rank the importance of nodes in networks based on link structures of nodes. The basic idea of PageRank algorithm is that the importance of a node is determined by the importance of their parents nodes and the number of their parents nodes. Therefore, by analyzing the quantity and quality of their parents nodes, PageRank algorithm can give a rough importance estimates for all nodes in networks.

In the classic PageRank algorithm, the importance of nodes can be defined as follows:

7 $$  PR (P_{i}) = \alpha \sum_{P_{j} \in \text{SCL}(P_{i})} \frac{1}{\mathrm{L}(P_{j})} PR (P_{j}) + (1- \alpha) \frac{1}{N}  $$

where *N* is the number of the nodes, and L(*P*_*j*_) is the number of outbound links for node *P*_*j*_, which belongs to the set of nodes that link to *P*_*i*_, also denoted by SCL(*P*_*i*_). *α* is a dampening factor set to 0.85 in this paper.

Equation 7 can be re-written in a matrix form as:

8 $$ PR = M \times PR  $$

where

9 $$ M = \alpha M_{1} + (1- \alpha) M_{2},\quad \alpha \in \,[\!0,1]  $$

and

10 $$\begin{array}{*{20}l} M_{1}(i,j) = \left\{ \begin{array}{cc} \frac{1}{\mathrm{L}(P_{j})},& if \ P_{j} \in \text{SCL}(P_{i}),\\ 0, & \text{otherwise} \end{array} \right. \end{array} $$

11 $$ M_{2} = \frac{1}{N} \mathbf{1}_{N \times N}  $$

We propose a modified PageRank algorithm to calculate the importance of nodes MPR, defined as follows:

12 $$\begin{array}{*{20}l} \tilde{MPR}^{k+1} = \hat{M} \times MPR^{k} \end{array} $$

Here the modified iterator matrix $\hat {M}$ is divided into two matrices:

13 $$ \hat{M} = \alpha \hat{M}_{1} + (1- \alpha) \hat{M}_{2}, \quad \alpha \in\, [\!0,1]  $$

where sparse hyperlink matrix $\hat {M}_{1}$ are generated from the weighted protein-protein interaction networks:

14 $$ \begin{aligned} &\hat{M}_{1}(i,j) \\ &= \left\{ \begin{array}{cc}\frac{\mathrm{W_{PPI}}(P_{i},P_{j})}{\sum_{P_{k} \in \text{SCL}(P_{i})}\mathrm{W_{PPI}}(P_{i},P_{k})},& if \ P_{j} \in \text{SCL}(P_{i}),\\ 0, & \text{otherwise} \end{array} \right. \end{aligned}  $$

and the reset probability matrix M2 comes from the prior importance of proteins:

15 $$ \hat{M}_{2}(i,j) = \frac{\text{IPSC}(P_{i})}{\sum_{k=1}^{N} \text{IPSC}(P_{k})}  $$

Finally, the importance of nodes is normalized as follows:

16 $$\begin{array}{*{20}l} MPR^{k+1} = \frac{\tilde{MPR}^{k+1}}{\left\| \tilde{MPR}^{k+1} \right\|} \end{array} $$

### Pearson correlation coefficient

Pearson correlation coefficient (PCC) is a popular method to measure linear correlation between two variables. Here we utilize PCC, derived from gene expression data, to calculate the importance of protein-protein interactions. Given gene expression data of two proteins, denoted by *X*=(*x*_1_,⋯,*x*_*m*_) and *Y*=(*y*_1_,⋯,*y*_*m*_), the importance of protein-protein interactions between the two proteins can be calculated as follows:

17 $$ {\displaystyle \begin{aligned} PCC\left(\mathrm{X},\mathrm{Y}\right)& =\frac{\mathrm{Cov}\left(X,Y\right)}{\sigma_X{\sigma}_Y}\\ &=\frac{\sum\nolimits_{i=1}^m\left({x}_i-\bar{x}\right)\left({y}_i-\bar{y}\right)}{\sqrt{\sum\nolimits_{i=1}^m{\left({x}_i-\bar{x}\right)}^2}\sqrt{\sum\nolimits_{i=1}^m{\left({y}_i-\bar{y}\right)}^2}}\end{aligned}} $$

Finally, the importance of each protein Pi, denoted as IPCC(Pi), is computed by summing up all weights of protein-protein interaction importance of protein *P*_*i*_:

18 $$ \text{IPCC} (P_{i}) = \sum_{P_{j} \in \text{SCL}(P_{i})} \text{PCC}(P_{i},P_{j})  $$

## Results and discussion

In this section, experiments are carried out to evaluate the effectiveness of our algorithm. We take advantage of three types of datasets, namely protein-protein interactions data, gene expression data and subcellular localization data, to predict essential proteins for Saccharomyces cerevisiae. We compare the performance of our algorithm SCP against other five methods (CIC, DC, NC, PeC, WDC) on real dataset of essential proteins. The results show that our method SCP outperforms the other five methods.

### Experimental data

#### Protein-protein interactions data

We downloaded protein-protein interaction networks from the Biogrid database (BIOGRID-3.2.111), which is a freely accessible database to provide physical and genetic interactions [28]. The network consists of 6304 proteins and 81,614 interactions between them.

#### Gene expression data

The gene expression data of yeast was obtained from the NCBI Gene Expression Omnibus website. This dataset was collected at 36 different times from 9335 probes (uploaded on April 14, 2011), since there is evidence that the expression of gene is periodic during metabolic cycle of Saccharomyces cerevisiae [29]. In total 6777 genes are present in the dataset, some of which have more than one expression profiles. For genes that have multiple expression profiles, we select the profile whose average is maximum.

#### Subcellular localization data

The COMPARTMENTS database [30] contains subcellular localization information from several data sources, such as literature, high-throughput microscopy-based screens, prediction from primary sequence and text mining. The dataset includes 819 subcellular compartments in total, which was downloaded on April 20, 2014.

#### Essential protein set

This set of essential proteins were downloaded from DEG [3], MIPS [31], SGD [32] and SGDP. It contains 1204 essential proteins in all.

### ROC curves

The proteins of Saccharomyces cerevisiae are classified into essential and nonessential proteins, so the prediction of essential proteins is actually a two-class classification problem. Hence, ROC curve is a proper metric to evaluate the performance of a binary classifier, plotted at different thresholds. In an ROC curve, the horizontal axis represents the values of false positive rate (FPR) and vertical axis represents the values of the true positive rate (TPR). The false positive rate is also known as specificity and the true positive rate is also known as sensitivity or recall. They are defined as follows:

19 $$\begin{array}{*{20}l} \text{FPR} = \frac{\text{FP}}{\text{FP}+\text{TN}} \end{array} $$

20 $$\begin{array}{*{20}l} \text{TPR} = \frac{\text{TP}}{\text{TP}+\text{FN}} \end{array} $$

where FP is the number of false positive, which means a prediction is positive and the actual value is negative. Conversely, FN is the number of false negative, which means the prediction is negative while the actual value is positive. Then TP is the number of true positive when both the prediction and actual value are positive. TN is the number of true negative when both the prediction and true value are negative.

**Fig. 1:**
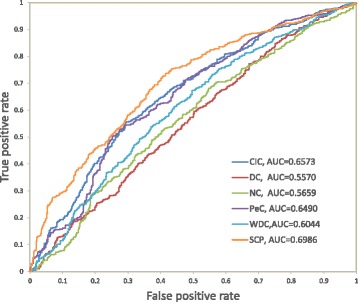
ROC curves of all methods

Furthermore, the size of the area under the curve, named AUC, is used to evaluate the performance of a binary classifier. Therefore, the larger the AUC value is, the better classifier is. In Fig. 1, ROC curves are plotted to analyze the top 1204 proteins ranked by all six algorithms, because our dataset contains 1204 essential proteins in total. As DC is a simple topological centrality algorithm, the AUC for DC is only 0.5570. Then NC is a method applying the edge-clustering coefficient to predict essential proteins, which achieves a litter better performance than DC. PeC and WDC have higher AUC values than DC and NC since they both incorporate gene expression data with PPI data to boost classification performance. CIC performs better than PeC, WDC, NC and DC, since it combines the subcellular localization information with other types of data. Lastly, our method SCP outperforms all the other five methods with a considerable margin. This shows the effectiveness of our fusion method.

### Analysis of essential proteins of top ranked proteins

**Fig. 2:**
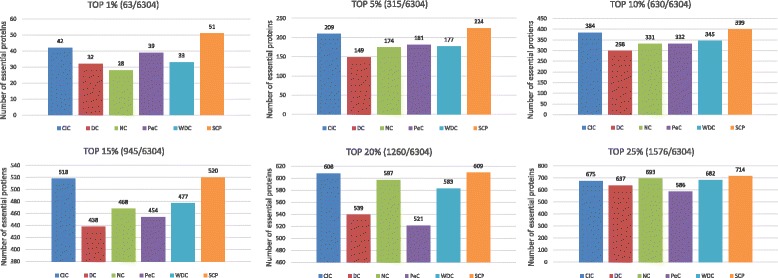
Number of essential proteins in ranked proteins

In this section, we attempt to visualize the proportion of essential proteins in top ranked proteins by all methods, including our method SCP and other five methods. First, we rank proteins by their importance scores in descending order computed by all six methods. Second, we select the top 1,5,⋯,25 percent of all 6304 proteins in their ranked order as essential protein candidates. Then we count the number of real essential proteins in these essential protein candidates according to the golden standard dataset of real essential proteins. The comparative results are shown in Fig. 2. From this figure, we can observe that the SCP outperforms all the other five algorithms on all six proportions of essential proteins.

In the Fig. 2, let us take the top 1% ranked proteins as an example: our method achieves considerable margin compared to other five methods (51 true essential proteins versus 42,32,28,39 and 33 for CIC, DC, NC, PeC and WDC respectively). In addition, Fig. 2 shows that DC and PeC performs better at top 1% and 5% than NC and WDC. However, from top 15 to 25%, the performances of NC and WDC are better than those of DC and PeC. The performance of CIC is good except at the top 25% ranked proteins, when it ranks fourth, and is only better than DC and PeC. In summary, our method achieves the best performances consistently at various percentage of top ranked proteins.

### Jackknife curves

**Fig. 3:**
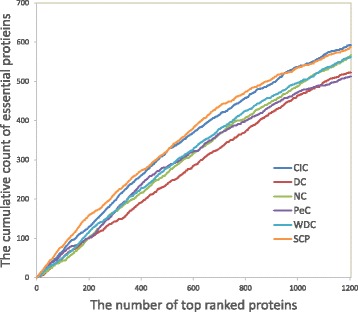
Jackknife curves of all methods

In this section, we compare our method with five other methods by the jackknife curves, which is proposed by Holman et al. [33] to show the ability to recover known essential proteins. The results are shown in Fig. 3. The horizontal axis of the jackknife curves represents the proteins ranked by scores of importance in descending order from left to right. In this section, we choose the top 1204 proteins of all the six methods to analyze the performance.The vertical axis is the cumulative count of essential proteins. Compared with other five methods, the AUC of our method is the largest. The Jackknife curves also reveal that the performance of our method SCP is better than the other methods.

### Precision-recall curves

In this section, we employ precision-recall (PR) curves to compare the performance of our method SCP with the other methods. The recall has been defined as the true positive rate (TPR) in “ROC curves” section. The precision is defined as follows:

21 $$ \text{Precision} = \frac{\text{TP}}{\text{TP}+\text{FP}}  $$

**Fig. 4:**
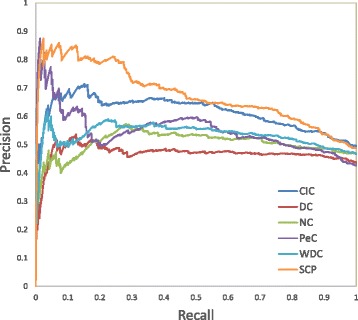
Precision-recall curves of all methods

To analyze a binary classification, precision is a measure of the proportion of results that are relevant to the query, and recall is a measure of the proportion of results relevant to the query that are successfully retrieved. If AUC is high, both precision and recall are high. High score of precision suggests the classifier achieves accurate results, while high recall indicates the classifier obtains a majority of all positive results. Because there are 1204 essential proteins in our dataset, we also plot PR curves to analyze the top 1204 proteins ranked by all six algorithms. It is shown in Fig. 4 that SCP achieves the best performance among all the methods.

### The analysis of links between top ranked proteins

**Fig. 5:**
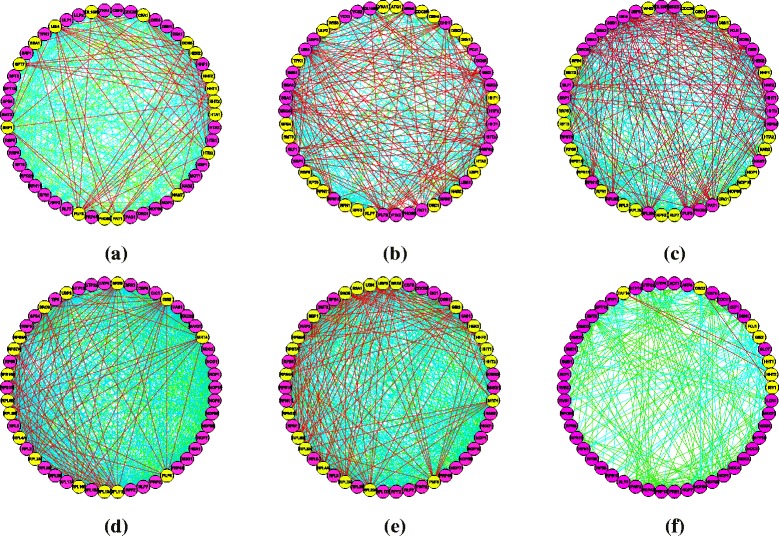
The comparative results of protein-protein interaction links by six methods. The figure shows the networks of the proteins ranked in top 50 by all six methods, and the links between them. The pink nodes represent the essential proteins, and the yellow nodes represent the nonessential proteins. Red, blue and green links represent Noness-Noness, Ess-Noness and Ess-Ess interactions respectively. **a** CIC. **b** DC. **c** NC. **d** PeC. **e** WDC. **f** SCP

In this section, we will do some further analysis of the links between top ranked proteins for all the methods. We construct small PPI networks based on the top 50 ranked proteins and the links depending on the whole yeast PPI networks. The results are shown in Fig. 5. Pink nodes represent essential proteins, while yellow nodes represent nonessential proteins identified by six methods. In this study, 43 essential proteins are obtained by our method SCP in the top 50 proteins, while for CIC, DC, NC, PeC, WDC, it is only 33, 22, 23, 34 and 28 respectively. Meanwhile, we analyze the links between top ranked proteins. As the number of links between top ranked proteins is different for various methods, we calculate the proportion of the links between essential proteins (Ess-Ess), between essential proteins and nonessential proteins (Ess-Noness), and between nonessential proteins (Noness-Noness). In Fig. 5, red, blue and green links represent Noness-Noness, Ess-Noness and Ess-Ess interactions respectively. From the Fig. 5, it is easy to find for SCP, the number of Noness-Noness interactions is much less than those of the other methods. For Ess-Ess and Ess-Noness interactions, it is not easy to distinguish the difference of all the methods as these kinds of links are too many. Therefore, in order to show more details of the comparison of SCP and other methods, many experiments are carried out shown in Table 1. It shows the proportions of Ess-Ess, Ess-Noness and Noness-Noness from top 100 to top 400 ranked proteins for all six methods. From the table, it shows SCP obtained the best performance of all the methods. For instance, in the top 100 ranked proteins, the proportion of Noness-Noness for our method is only 4.11%, which is much lower than other methods, while the proportion of Ess-Ess for our method is up to 63.58%, which is the highest of all the methods.

**Table 1:** Analysis of link proportion

Top	Link	CIC	DC	NC	PeC	WDC	SCP
100	Ess-Ess	44.64%	27.82%	18.34%	42.22%	26.43%	**63.58%**
	Ess-Noness	43.21%	45.86%	45.52%	35.91%	44.92%	32.31%
	Noness-Noness	12.15%	26.32%	36.14%	21.87%	28.64%	**4.11**%
200	Ess-Ess	45.91%	26.78%	23.86%	35.74%	34.03%	**66.05%**
	Ess-Noness	41.70%	47.80%	42.88%	35.94%	41.50%	28.21%
	Noness-Noness	12.39%	25.33%	33.27%	28.32%	24.46%	**5.74%**
300	Ess-Ess	45.74%	23.58%	30.33%	37.20%	35.02%	**53.90%**
	Ess-Noness	41.68%	47.01%	42.62%	36.18%	40.96%	35.84%
	Noness-Noness	12.58%	29.41%	27.05%	26.62%	24.02%	**10.26%**
400	Ess-Ess	46.15%	23.74%	30.89%	39.58%	35.35%	**51.23%**
	Ess-Noness	40.94%	46.22%	42.36%	36.39%	40.96%	37.20%
	Noness-Noness	12.92%	30.04%	26.75%	24.04%	23.70%	**11.56%**

(Optimal values are denoted by boldface)

### The analysis of parameter *λ*

In this section, we discuss the selection of parameter *λ*. As the prediction of essential proteins is an unsupervised learning procedure, we can’t learn a best parameter *λ* from the data. Therefore, we only choose *λ*∈{0,0.5,1} to analyze the performance of our algorithm SCP. In reality, when *λ*=0, the results of SCP only come from IPCC. Conversely, the results will only be calculated by MPR when *λ*=1. In this paper, we chose *λ* as 0.5, which means the results of SCP integrate MPR and IPCC. In order to compare the performance of the method on various *λ*, we calculate the number of essential proteins at different top percentages of ranked proteins (top 1%, 5%, 10%, 15%, 20%, 25%). From Table 2, it demonstrates that when *λ*=0.5, SCP obtains the best performance. Therefore, in this paper the parameter *λ* is set as 0.5. As a result, SCP successfully integrates the results of MPR and IPCC and has achieved a great boost on the performance of essential proteins prediction.

**Table 2:** Number of essential proteins in top ranked proteins from SCP on various value of *λ*

*λ*	1%	5%	10%	15%	20%	25%
0	45	173	335	437	521	589
0.5	**51**	**224**	399	**520**	**609**	**714**
1	49	216	**403**	517	603	700

(Optimal values are denoted by boldface)

### The analysis of the performance of CIC and SCP

In this section, we will analyze the performance of CIC and SCP. Both CIC and SCP utilize the subcellular localization information to predict the essential proteins, while SCP also use the information of the gene expression data. Therefore, we will compare CIC with modified PageRank (MPR), part of our method SCP, which only uses the subcellular localization information as CIC does to predict essential proteins. The results are shown in Table 3. Although the performance of MPR is worse than SCP, MPR achieves better performance than CIC in most cases, except for top 15 and 20 percentages, where the number of essential proteins identified by MPR is a little less than those does by CIC.

**Table 3:** Number of essential proteins in top ranked proteins identified by CIC, MPR and SCP

Method	1%	5%	10%	15%	20%	25%
CIC	42	209	384	518	608	675
MPR	49	216	**403**	517	603	700
SCP	**51**	**224**	399	**520**	**609**	**714**

(Optimal values are denoted by boldface)

## Conclusion

Essential proteins are crucial to the development and survival of life. Many computational methods are proposed to detect essential proteins based on biological and topological features of proteins. In our study, we also found that integration of information from multiple sources can boost the identification of essential proteins. Specifically, the utilization of subcellular localization information can make a remarkable contribution to the prediction of essential proteins. In this paper, a SCP method is proposed, which integrates the ranking function by a modified PageRank algorithm with weighted subcellular localization with Pearson correlation coefficient based on gene expression data. Several experiments are carried out to compare the performance of SCP with five other methods in identification of essential proteins. Experimental results show that our method SCP performs the best among all six methods.
